# Supplementary material for: Effects of Trichoderma harzianum combined with Phanerochaete chrysosporium on lignin degradation and humification during chicken manure and rice husk composting
Source: Front Microbiol. 2025 Feb 28;16:1515931. doi: 10.3389/fmicb.2025.1515931 (PMC11906335; doi:10.3389/fmicb.2025.1515931)
Supplement: Supplementary file 1 [file Data_Sheet_1.zip › 6.Function/CK0_BFF/FunGuild/l2/metastats/Ericoid_Mycorrhizal.CK0_BFF.metastats.pdf]

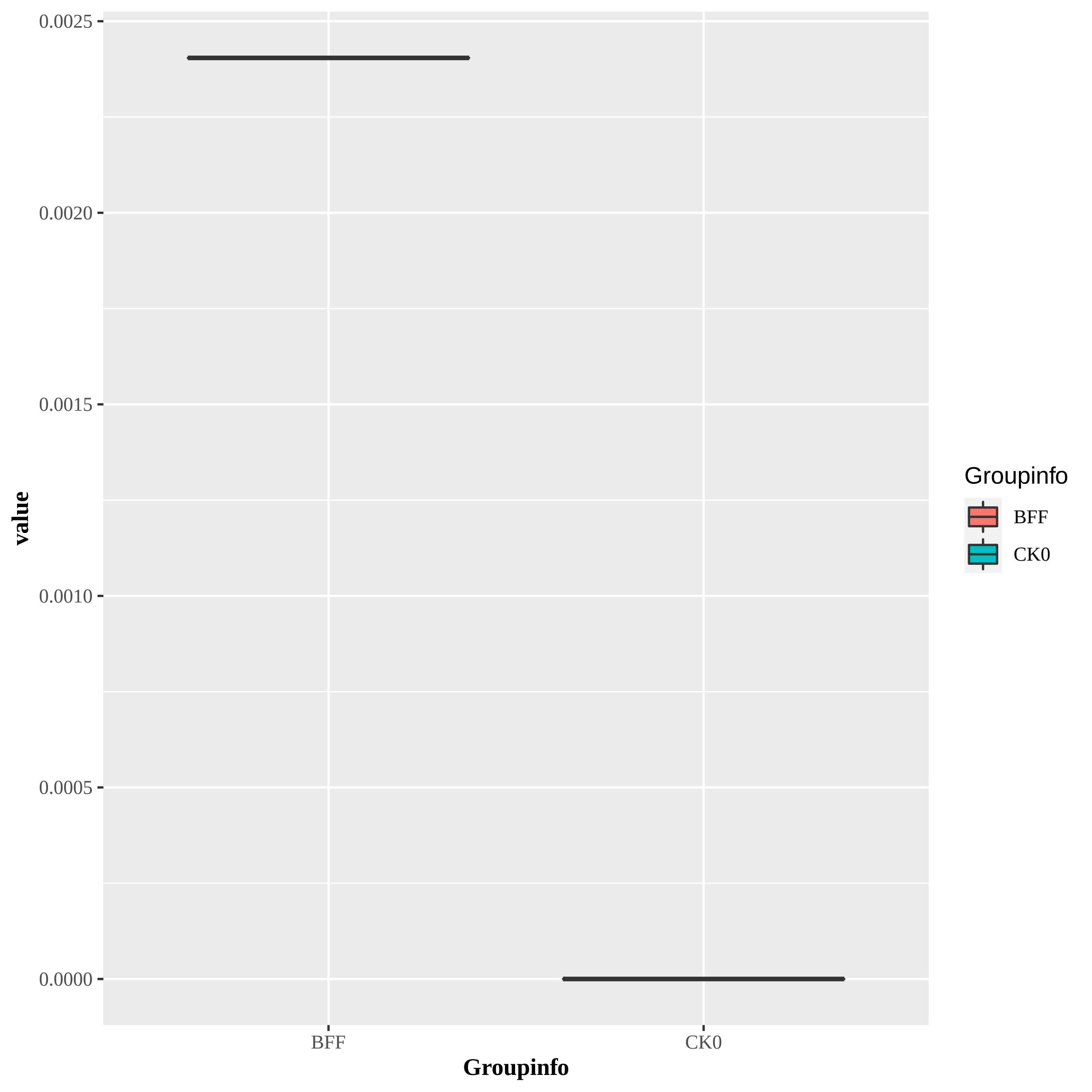
0.0025
0.0020
0.0015
0.0010
0.0005
0.0000
value
BFF
CK0
Groupinfo
Groupinfo
BFF
CK0
